# Synthesis of Aluminum-Based Metal–Organic Framework (MOF)-Derived Carbon Nanomaterials and Their Water Adsorption Isotherm

**DOI:** 10.3390/nano13162351

**Published:** 2023-08-16

**Authors:** Dasom Jeong, Seong Cheon Kim, Taeseop An, Dongho Lee, Haejin Hwang, Siyoung Q. Choi, Jeasung Park

**Affiliations:** 1 Green and Sustainable Materials R&D Department, Korea Institute of Industrial Technology (KITECH), Cheonan 31056, Republic of Korea; jdasom0130@kitech.re.kr (D.J.); grey@kitech.re.kr (S.C.K.); ats2660@kitech.re.kr (T.A.); 2 Department of Materials Science and Engineering, INHA University, Incheon 22212, Republic of Korea; hjhwang@inha.ac.kr; 3 Department of Chemical and Biomolecular Engineering, Korea Advanced Institute of Science and Technology (KAIST), Daejeon 34141, Republic of Korea; sqchoi@kaist.ac.kr; 4 Process R&D Center, Hanwha Solutions R&D Institute, Daejeon 34128, Republic of Korea; dhlee@hanwha.com; 5 KAIST Institute for the Nanocentury, Korea Advanced Institute of Science and Technology (KAIST), Daejeon 34141, Republic of Korea

**Keywords:** metal–organic framework (MOF), MOF-derived carbon (MDC), CAU-10-H, aluminum-fumarate, water adsorption, isotherm model

## Abstract

The characteristics of water vapor adsorption depend on the structure, porosity, and functional groups of the material. Metal–organic framework (MOF)-derived carbon (MDC) is a novel material that exhibits a high specific area and tunable pore sizes by exploiting the stable structure and porosity of pure MOF materials. Herein, two types of aluminum-based MOFs were used as precursors to synthesize hydrophobic microporous C-MDC and micro-mesoporous A-MDC via carbonization and activation depending on the type of ligands in the precursors. C-MDC and A-MDC have different pore characteristics and exhibit distinct water adsorption properties. C-MDC with hydrophobic properties and micropores exhibited negligible water adsorption (108.54 mgg^−1^) at relatively low pressures (*P*/*P*_0_~0.3) but showed a rapid increase in water adsorption ability (475.7 mgg^−1^) at relative pressures of about 0.6. A comparison with the isotherm model indicated that the results were consistent with the theories, which include site filling at low relative pressure and pore filling at high relative pressure. In particular, the Do–Do model specialized for type 5 showed excellent agreement.

## 1. Introduction

Porous materials have received considerable attention in various fields, including in the environmental and energy sectors, owing to their potential for addressing a range of environmental issues [1]. In particular, porous materials with excellent physical water adsorption capabilities and high chemical stability have attracted attention for applications in adsorption cooling, desalination, and desiccant air-cooling systems [2,3,4]. The performance of an adsorbent is determined by its ability to adsorb water at different pressure ranges, depending on the specific application requirements [5].

Metal–organic frameworks (MOFs) are a class of potential materials used as adsorbents. They are porous coordination polymers composed of inorganic metal ions or metal clusters and organic ligands linked together to form three-dimensional crystalline frameworks. MOFs exhibit high chemical stability owing to high surface area and well-ordered structure and have advantageous properties, such as tunable porosity and modified functional groups [6,7,8]. Materials used as adsorbents must have an appropriate pressure range to suit the intended application. Various studies have reported the modification of functional groups in MOFs for adjusting the relative pressure range by exploiting their hydrophobic or hydrophilic properties [9,10]. However, controlling the relative pressure range has limitations because the range is not very wide, and it can adversely affect the water adsorption capacity [11].

MOFs can be transformed into more stable nanomaterials such as carbon materials, metal oxides, and hybrid materials, as required. MOF-derived materials can be manufactured without additional templates. Moreover, the size and volume of their pores can be freely controlled, and hence, they can be used for doping and hybridization [12,13,14]. Owing to these characteristics, MOF-derived materials have received considerable attention and have been widely studied in the fields of electrochemical energy storage and conversion devices, including supercapacitors, batteries, and fuel cells [15,16]. Among them, MOF-derived carbon (MDC) is a novel carbon material that utilizes the stable structure and porosity of pure MOFs and exhibits a high specific surface area and tunable porosity [17]. The properties of the MDC depend on the structure of the precursor material, the MOF. In addition, various structures can be formed using different heat treatments and activation conditions, although the underlying reasons for this structure-forming phenomenon are not yet fully understood. Hence, MDCs have garnered considerable attention in the fields of electrochemistry and energy, where excellent electrical conductivity and porous carbon are necessary [15,18,19]. While various studies have investigated the water adsorption properties of biomass-derived activated carbon, there is a lack of research on the water adsorption properties of MDCs [20,21,22,23]. The properties and composition of biomass-derived activated carbon can vary due to natural environmental factors, making it difficult to control the pore size. However, MDCs can be manufactured with consistent properties and compositions, and thus have a competitive advantage for commercial use.

According to the International Union of Pure and Applied Chemistry (IUPAC), there are seven types of isotherm water vapor adsorption curves [24]. Types 1, 2, 4, and 6 represent the water adsorption properties of hydrophilic materials that exhibit adsorption at relatively low pressures. In contrast, types 3, 5, and 7 represent the water adsorption properties of hydrophobic materials that do not exhibit water adsorption at low relative pressures and show adsorption characteristics as the relative pressure increases [25]. The adsorption mechanism also varies depending on pore size. Carbon materials with micropores exhibit a sharp increase in water adsorption in the relative pressure range of 0.6, whereas carbon materials with mesopores demonstrate high water adsorption characteristics at a relative pressure of ~0.8, and materials with macropores show high water adsorption properties at relative pressures above 0.9 [26,27].

CAU-10-H is a MOF composed of aluminum clusters and isophthalic acid organic ligands. CAU-10-H exhibits excellent chemical and water stability. Because of the low cost of aluminum salts and isophthalate ligands, it is an excellent material for commercial use. CAU-10-H comprises helical chains of *-cis* corner-sharing AlO_6_ octahedra interconnected by isophthalate ions, forming a three-dimensional structure with one-dimensional channels of spherical shape measuring 4.0 × 7.0 Å [9,28,29,30].

Aluminum-fumarate (Al-fu) is a MOF composed of aluminum clusters and fumaric acid organic ligands. The manufacturing costs of Al-fu are low because aluminum is used as the metal cluster, similar to that in CAU-10-H. Al-fu exhibits high chemical and water stability and excellent water adsorption capabilities. Al-fu comprises helical chains of *-trans* corner sharing AlO_6_ octahedra interconnected by fumarate ions, forming a three-dimensional structure with one-dimensional channels of spherical shape measuring 5.7 × 6.0 Å [31,32].

Large-scale synthesis of the two aluminum-based MOFs (CAU-10-H and Al-fu) is feasible owing to the low cost of ligands and metal salts, along with an eco-friendly synthesis process using water as the solvent. Hence, they have a high potential for commercial development [S.1, S.2].

In this study, we synthesized MDCs via carbonization and activation using two aluminum-based MOFs as pure materials. The synthesized MDCs exhibited different structures and pore characteristics owing to the differences in the structure and elements of the precursor MOFs. Differences in pore distribution led to distinct water adsorption properties governed by the water adsorption mechanism. We analyze the pore structure, physical and chemical properties, and their correlation with the water adsorption mechanism and demonstrate the potential of MDCs as water adsorbents.

## 2. Materials and Methods

### 2.1. Materials

The reagents used for the synthesis were aluminum sulfate 14–18 H_2_O (51.0–57.5%, SAMCHUN, Pyeongtaek-si, Republic of Korea), sodium aluminate (38.0–42.0% Na_2_O, 51.0–55.0% Al_2_O_3_, JUNSEI, Tokyo, Japan), isophthalic acid (99.0%, SAMCHUN, Seoul, Republic of Korea), sodium hydroxide (98.0%, SAMCHUN, Republic of Korea), fumaric acid (99.0%, SAMCHUN, Republic of Korea), urea (99.0%, SAMCHUN, Republic of Korea), ethanol (94.0%, DUKSAN, Seoul, Republic of Korea), and deionized water.

### 2.2. Synthesis of CAU-10-H

CAU-10-H was synthesized via a procedure described in the literature with water as the solvent [33]. The CAU-10-H was formed via the coordination bonding of aluminum clusters and organic ligands, with sodium hydroxide acting as a modulator.

Sol. 1: 3.6 g (0.090 mol) of sodium hydroxide was added to 90 mL of deionized water in a 200 mL beaker and dissolved for 1 h. Subsequently, 7.476 g (0.045 mol) of isophthalic acid was added to the solution and dissolved at 35 °C for 3 h. Sol. 2: in another 100 mL beaker, 11.263 g (0.017 mol) of aluminum sulfate was dissolved in 33.75 mL of deionized water. Sol. 3: in another 100 mL beaker, 0.922 g (0.011 mol) of sodium aluminate was dissolved in 22.5 mL of deionized water. Solution 1 and 7.5 mL ethanol were mixed in a three-neck flask and stirred for 10 min. Thereafter, solutions 2 and 3 were added simultaneously, and the mixture was refluxed in an oil bath of 160 °C for 20 h. The synthesized solution was washed twice with deionized water and ethanol, filtered using a paper filter, and dried in a vacuum oven at 80 °C overnight to obtain CAU-10-H.

### 2.3. Synthesis of Aluminum Fumarate MOF

Al-fu was synthesized via a reflux reaction. Al-fu was formed via the coordination bonding of aluminum clusters and the organic ligand, fumaric acid, with urea acting as a modulator.

Next, 78.64 g (0.125 mol) of aluminum sulfate and 22.48 g (0.375 mol) of urea were added to 400 mL of deionized water in a 600 mL of beaker and stirred until fully dissolved. Next, 28.96 g (0.250 mol) of fumaric acid was added to the solution and stirred for 30 min at 25 °C. The mixture was then refluxed in an oil bath of 80 °C for 24 h, followed by refluxing at 110 °C for another 24 h. The resulting solution was washed three times with deionized water, filtered using a paper filter, and dried in a vacuum oven at 80 °C overnight to obtain Al-fu.

### 2.4. Synthesis of MDCs

The CAU-10-H and Al-fu synthesized herein were used as precursors for synthesizing MDCs. C-14PF-50 (HANTECH, Ulsan, Republic of Korea) semi-tube furnace with a tube size of 50 × 46 × 250 mm was used for carbonization, and nitrogen gas (99.999%) was supplied at a flow rate of 200 cc/min. Sodium hydroxide (98.0%, SAMCHUN, Republic of Korea) was used as the wet etching solution for the carbonized CAU-10-H and carbonized Al-fu.

To synthesize CAU-10-H MDC, 1 g of CAU-10-H in a ceramic boat was placed in the tube furnace. The furnace is heated at a rate of 5 °C per minute until it reaches 900 °C, and then the sample was carbonized at 900 °C for 3 h under a nitrogen atmosphere. The carbonized sample was cooled to room temperature and was poured into 100 mL of 8 M sodium hydroxide solution and activated by stirring for 1 h at 95 °C. The activated sample was washed thoroughly with deionized water, filtered using a paper filter, and dried overnight in a vacuum oven at 80 °C to obtain CAU-10-H MDC.

Al-fu MDC was synthesized via carbonization of Al-fu at 800 °C for 3 h and activation by stirring for 1 h at 90 °C in 10 M sodium hydroxide solution. The other synthesis conditions were identical to those used for the synthesis of CAU-10-H MDC.

Hereafter, the CAU-10-H MDC is referred to as C-MDC, and the Al-fu MDC is referred to as A-MDC.

### 2.5. Material Characterization

The crystal structures of the MOFs and MDCs were examined via X-ray diffraction (XRD) analysis with Cu-Kα radiation, current of 30 mA, and voltage of 40 kV using a LabX XRD-6100 (Shimadzu, Kyoto, Japan); the scan mode was continuous with 2θ in the range 3–80° and the measurement speed was 10°/min. The elemental composition and chemical bonding states of the sample surfaces were investigated via X-ray photoelectron spectroscopy (XPS) using a K-Alpha instrument (Thermo Fisher Scientific, Waltham, MA, USA) with an Al X-ray source (1486.7 eV). The microstructure and distribution of components in the MOFs and MDCs were analyzed via FE-SEM and EDS using JSM-6701F (JEOL, Tokyo, Japan). The carbon, nitrogen, hydrogen, and oxygen contents of the MOFs and MDCs were examined via elemental analysis (EA) using a Flash*Smart*^TM^ Elemental Analyzer (Thermo Fisher Sciencific, Waltham, MA, USA). Each sample was measured three times and the average value was used for quantitative calculations. The presence of functional groups on the MDC sample surfaces was investigated by measuring the infrared absorption via FTIR analysis. A Nicolet 6700 (Thermo Fisher Scientific, Waltham, MA, USA) was used for the FTIR analysis, and measurements were performed in the attenuated total reflectance measurement mode in the range of 4000–660 cm^−1^. The pore size, volume, and distribution of the MOFs and MDCs were determined via nitrogen gas adsorption (77 K) analysis using a BELSORP MAX (MicrotracBEL, Osaka, Japan). Before the analysis, all samples were pretreated under vacuum conditions at 180 °C for 8 h. TEM and EDS analyses were conducted using a Tecnai G2 G30 S-Twin (FEI, Naples, Italy). Raman spectroscopy was conducted at a wavelength of 532 nm using a LabRAM Revolution (HORIBA, Kyoto, Japan).

### 2.6. Water Vapor Adsorption Evaluation and Isotherm Model

The water adsorption capacities of the MOFs and MDCs were investigated via water adsorption analyses using MicrotracBEL BELSORP MAX (MicrotracBEL, Osaka, Japan) under isothermal conditions at 298 K and 308 K. The adsorption and desorption ranges were set at relative pressures (*P*/*P*_0_) of 0–0.990 and 0.300–0.990, respectively. For pressure measurement, 5 sensors with a range of 133 kPa (1000 Torr) and 2 sensors with a range of 1.33 kPa (10 Torr) were used, each having an error range of 0.15% and 0.5%, respectively.

Two theoretical models were used to validate the water sorption properties of the MDCs obtained from the water adsorption analyses and further expand their practical application range. The first model used was the Henry–Sips model, expressed as follows: [34]

(1) $$q=\beta K_{H}\left(\frac{p}{p_{0}}\right)+\left(1-\beta \right)\frac{q_{m}{\left(K_{s}p/p_{0}\right)}^{1/n}}{1+{\left(K_{s}p/p_{0}\right)}^{1/n}}$$

(2) $$K=K_{0}exp\left(\frac{-∆H}{RT}\right)$$

(3) $$\beta =exp\left(-\alpha \frac{p}{p_{0}}\right),\;n=A+\frac{B}{T}$$

where *q* is the equilibrium adsorption capacity, *q_m_* is maximum adsorption capacity, *p* is the equilibrium pressure (in kPa), *p*_0_ is the saturation pressure (in kPa), *K_H_* is the Henry constant, *K_s_* is the Sips constant, and *n* is the degree of surface nonhomogeneity.

The second model is the Do–Do model, expressed by the following equation [35,36]:

(4) $$\theta =f\frac{K_{f}x\left\{1-\left(1+\beta \right)x^{\beta }+\beta x^{\beta +1}\right\}}{\left(1-x\right)\left\{1+\left(K_{f}-1\right)x-K_{f}x^{\beta +1}\right\}}+\left(1-f\right)\frac{K_{\mu }x^{\alpha }}{1+K_{\mu }x^{\alpha }};\beta \geq \alpha .$$

The *R*^2^ values for the two modeling curves were calculated as follows.

(5) $$R^{2}=1-\frac{\sum_{i=1}^{m}{\left(q_{meas,i}-q_{cal,i}\right)}^{2}}{\sum_{i=1}^{m}{\left(q_{meas,i}-{\bar{q}}_{meas}\right)}^{2}}$$

(6) $${\bar{q}}_{meas}=\frac{1}{m}\sum_{i=1}^{m}q_{meas,i}$$

where *m* is the number of measured isotherms.

The isosteric enthalpy of adsorption (Δ*H_ads_*) was calculated using the following equation [37]:

(7) $$∆H_{ads}=RT^{2}{\left\{\frac{\partial lnP}{\partial T}\right\}}_{q^{a}}=-R{\left\{\frac{\partial lnP}{\partial \frac{1}{T}}\right\}}_{q^{a}}$$

where *q^a^* denotes the adsorption capacity at a given temperature, and the enthalpy of adsorption at that point is calculated.

Repeated water adsorption and desorption analyses were conducted using a DVS Intrinsic (Surface Measurement Systems, Wembley, UK) to test the water stability of the MDCs. Prior to the analysis, the samples were dried with N_2_ gas (99.999%) at a flow rate of 200 cc/min for 300 min until the dm/dt value reached 0.001. The cutoff condition for each step was set at dm/dt = 0.005, and the relative humidity range was set at 10–90%. The water adsorption and desorption cycle tests were repeated 100 times.

## 3. Results and Discussion

**Figure 1:**
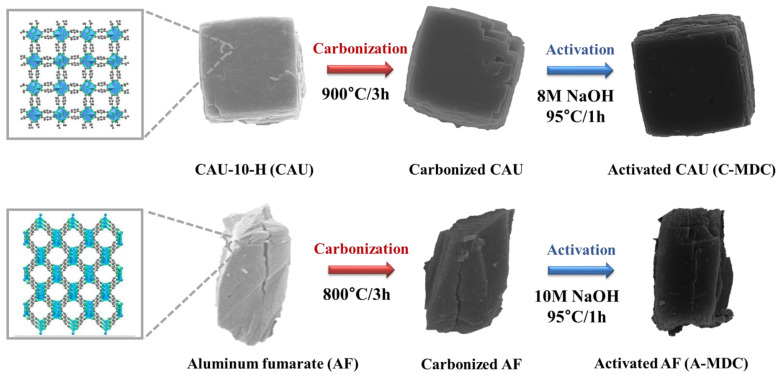
The schematics of fabrication of Al-based MOF-derived carbon.

Figure 1 depicts a schematic of the synthesis of MDC, which was derived from a precursor using MOF as a template via carbonization and activation. MDC maintains its morphology without significant changes even after heat treatment, which is essential for utilizing the porous properties of MOF.

**Figure 2:**
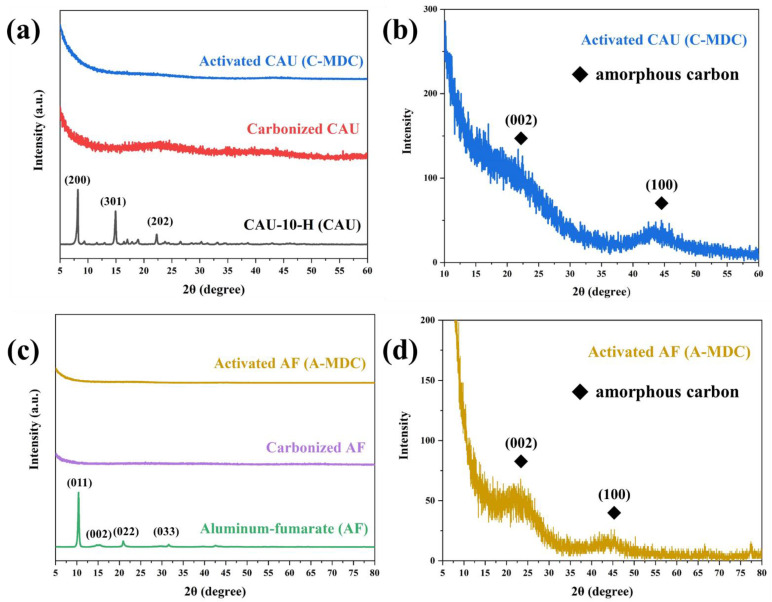
XRD patterns of MOF and MDC. CAU-10-H (CAU), carbonized CAU, and activated CAU (C-MDC) (**a**); C-MDC (**b**); Al-fu (AF), carbonized AF, and activated AF (A-MDC) (**c**); A-MDC (**d**).

Figure 2 shows the XRD patterns of CAU-10-H (which was used as a precursor) and C-MDC (which was synthesized via carbonization and activation). As shown in Figure 2, (a) sharp peaks were observed at 2 theta angles of 8.2°, 9.2°, 11.6°, 12.3°, 15°, 16.4°, 17°, 17.5°, 18.4°, 18.5°, 18.9°, and 22.3°, which correspond to the (200), (101), (220), (211), (301), (400), (231), (330), (240), and (202) planes of CAU-10-H [38]. As shown in Figure 2b, the sharp peaks of CAU-10-H were not observed after carbonization and the noise of the peaks increased, whereas broad peaks at approximately 22° and 43° were observed. The broad peak at 22° corresponds to an amorphous carbon structure, whereas the broad peak near 43° corresponds to a graphitic structure (JCPDS No. 41-1487). These results suggest that the bond between the aluminum clusters and organic ligands in the precursor was broken during carbonization, leading to the dispersion of pure aluminum metal inside the structure and the formation of an unstable amorphous carbon structure. The increase in the noise of the peak is attributed to the impurities generated during carbonization covering the surface. The intensity of the broad peak at 22°, corresponding to amorphous carbon, and the broad peak near 43°, corresponding to the graphitic structure, were higher, and the noise of the peaks was reduced after activation via wet etching. These results are attributed to the removal of impurities that remained on the surface during activation.

As shown in Figure 2c, sharp peaks were observed at 2 theta angles of 10.4°, 15°, 21°, 32°, and 43°, which correspond to the (011), (002), (022), (033), and (212) planes of Al-fu [39]. As shown in Figure 2d, similar to that in the C-MDC XRD pattern, diffraction peaks corresponding to the original crystal structure of MOF were not observed due to the decomposition of the aluminum clusters and organic ligands of the precursor during heat treatment, and an unstable amorphous carbon structure was formed. After activation via wet etching, impurities were removed, and broad peaks were observed at 22° and 43°. Unlike CAU-10-H, which has a benzene ring bond in its ligands, Al-fu has a different ligand structure. Hence, the (002) plane corresponding to the amorphous carbon structure was more distinct than the (101) plane corresponding to the graphitic structure. These results indicate that the structure of the MDC produced through heat treatment and etching can vary depending on the type of ligand.

**Figure 3:**
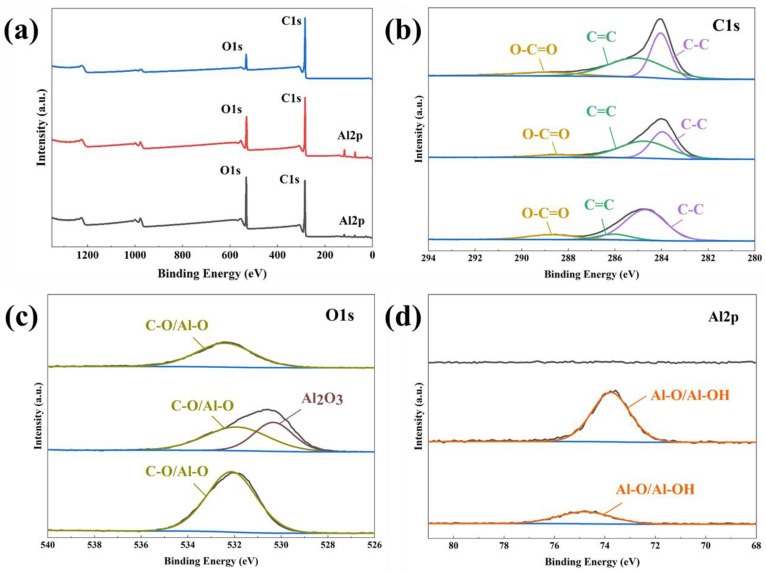
Results of XPS analyses of CAU-10-H and its derivatives. Survey scan spectrum (**a**) and high-resolution XPS scan spectrum over C1s (**b**); O1s (**c**); and Al2p spectra. The black line indicates the absence of any peaks (**d**).

Table 1 and Figure 3 present the results of the XPS analysis of the surface elements and chemical bonding of CAU-10-H and its derivatives produced via carbonization and activation. Figure 3b shows the carbon bonding state, wherein the peaks at 284.88 and 286.08 eV, respectively, indicate the presence of sp^2^ and sp^3^ carbon, in the ligands [40]. The peak at 288.68 eV indicates the bond between carbon and oxygen in the framework. The carbonization led to a partial cleavage of the sp^2^ carbon bonds and a relative increase in the proportion of sp^3^ carbon. The proportion of sp^3^ carbon was higher than that of the precursor even after the removal of impurities during activation. Figure 3c shows the oxygen bonding states, wherein the peak at 532.18 eV represents the carbon–oxygen bonding. The new peak at 530.38 eV corresponds to aluminum oxide formed during carbonization [41,42]. Aluminum oxide can block the surface and pores, potentially hindering the full utilization of pore characteristics. However, all impurities were removed after the activation process, as confirmed by the absence of an aluminum oxide peak in Figure 3c. As a result, it was confirmed that the formation of a carbonaceous structure on the surface of the C-MDC, wherein the proportion of carbon was 90.35 (Table 1, Figure 3a).

**Figure 4:**
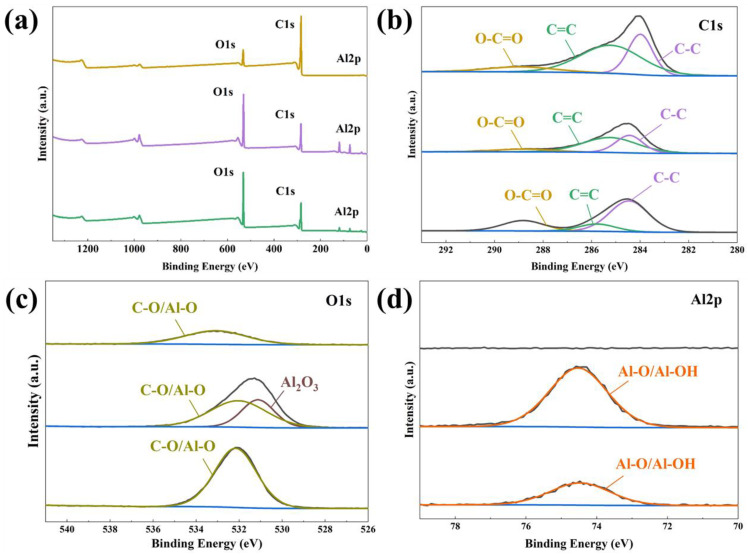
Results of XPS analysis of Al-fu and its derivatives. Survey scan spectrum (**a**) and high-resolution XPS scan spectrum over C1s (**b**); O1s (**c**); and Al2p spectra. The black line indicates the absence of any peaks (**d**).

Table 2 and Figure 4 present the results of XPS analysis of the surface elements and chemical bonding of Al-fu and its derivatives produced via carbonization and activation. The peaks at 284.08 eV and 285.18 eV, shown in Figure 4b, correspond to sp^2^ and sp^3^ carbon, respectively, as the ligands consist of both single and double carbon bonds [43]. The peak at 288.68 eV indicates the bond between carbon and oxygen in the framework. However, compared to CAU-10-H, the carbonization of Al-fu resulted in a significant reduction in sp^2^ carbon bonding, leading to a remarkable increase in the proportion of sp^3^ carbon. Furthermore, even after the removal of impurities during activation, the proportion of sp^3^ carbon remained higher than that in the precursor. These differences in recombination suggest that the structure of A-MDC may be more flexible than that of C-MDC, which may affect the pore characteristics. As shown in Figure 4c, the peak at 532.08 eV corresponds to the bond between carbon and oxygen. The new peak at 531.08 eV indicates the formation of aluminum oxide during carbonization [44,45]. All these impurities were removed during activation, as evidenced by the absence of the aluminum oxide peak in Figure 4d. As a result, it was confirmed that the formation of a carbonaceous structure on the surface of the A-MDC, wherein the proportion of carbon was 89.32% (Table 2, Figure 4a). Based on these results, it is anticipated that the activation process will secure maximum pore volume by removing impurities such as aluminum metals. Additionally, increasing carbon purity is expected to enhance water adsorption capability.

**Figure 5:**
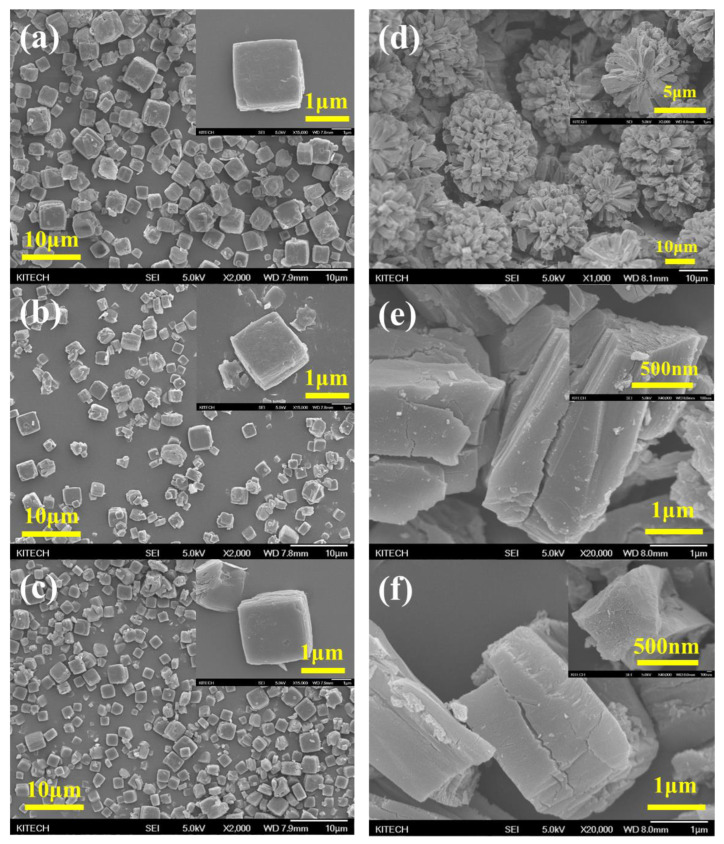
FE-SEM images of CAU-10-H, Al-fu, and their derivatives. CAU-10-H (CAU) (**a**); carbonized CAU (**b**); and activated CAU (C-MDC) (**c**); Al-fu (AF) (**d**); carbonized AF (**e**); and activated AF (A-MDC) (**f**).

Figure 5 shows the FE-SEM images of CAU-10-H, Al-fu, and their derivatives. As shown in Figure 5a, CAU-10-H exhibits a cubic morphology with an average particle size of 3 μm. As shown in Figure 5b,c, no significant morphological changes were observed after carbonization and activation. The morphology of the MDC obtained from the precursor MOF remained largely intact, demonstrating that the inherent porosity of the MOF structure was preserved.

The Al-fu synthesis method used herein was different from previously reported methods. Hence, significant differences were observed between the morphologies of Al-fu synthesized in this study and those reported in the literature. As shown in Figure 5d, rectangular particles with a size of 1.5 × 2.5 μm aggregated to form a flower-like shape, which is attributed to the controlled nucleation and nucleation growth rate in our synthesis method. Resting for 24 h before the reaction at a temperature lower than the reaction temperature makes nucleation growth delay and allows sufficient nucleation. The temperature was subsequently increased to initiate nucleation growth after sufficient nucleation. Thus, Al-fu with a uniform particle size was synthesized. As shown in Figure 5e, the particles that constituted the flower-like shape separated into individual entities after carbonization; however, the change in morphology was not significant. In addition, the morphology of the precursor did not change after activation (Figure 5f). However, compared to C-MDC, cracks were observed in the morphology of A-MDC after carbonization and activation.

The elemental compositions of CAU-10-H and its derivatives, obtained via EA and EDS, are presented in Table 3. Structural changes during MDC synthesis were predicted using the EA analysis results. The proportion of C, N, H, and O in carbonized CAU was 23.81 wt.% lower than that in CAU-10-H. This is expected because the cleavage of MOF bonds results in an increase in the proportion of aluminum. In addition, the proportion of carbon increases significantly owing to the removal of aluminum during activation. The results of EDS analysis similarly indicate that the proportions of aluminum and oxygen increase after carbonization. This suggests that the broken oxygen bonds combine with aluminum to form aluminum oxide, which is expected to be freely dispersed on the surface. Aluminum oxides are removed upon activation, and the EDS results indicate a higher proportion of carbon, which is consistent with the EA results. Both EA and EDS can be used for quantitative analysis of elements; however, EDS can only analyze the elements present on the surface, and hence, there may be some differences in the obtained proportions.

The elemental compositions of Al-fu and its derivatives are shown in Table 4. The results are consistent with the results of the analyses of CAU-10-H and its derivatives. The EA results confirmed that the increased aluminum generated during carbonization was largely removed via activation, resulting in a higher carbon ratio in the final product. EDS analysis indicated that A-MDC was primarily composed of carbon, with aluminum and oxygen present on the surface. The aluminum and oxygen on the surface formed aluminum oxide during carbonization, which was removed during activation. These results suggest that carbon is the primary constituent element of A-MDC.

**Figure 6:**
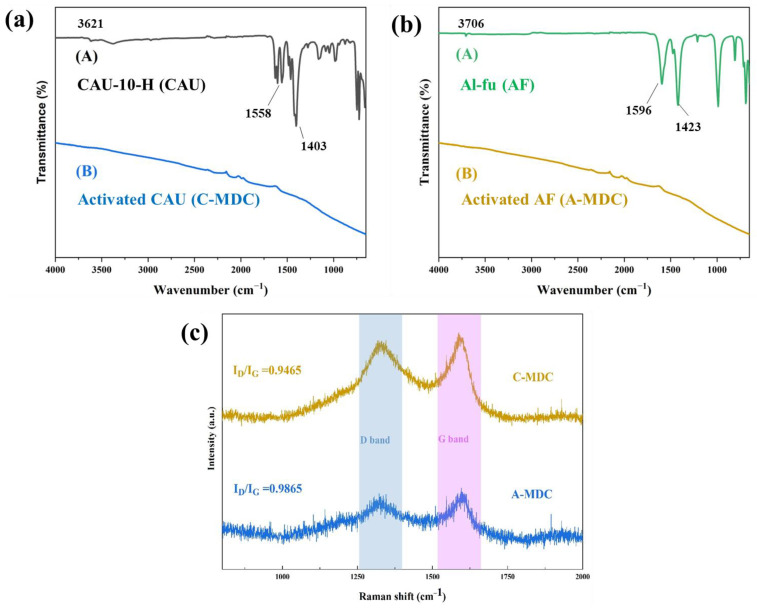
FT-IR results of CAU-10-H and C-MDC (**a**); FT-IR results of Al-fu and A-MDC; (**b**), Raman spectroscopy of C-MDC and A-MDC (**c**).

Fourier-transform infrared (FT-IR) analysis was conducted to identify the organic ligands of the MOF and the functional groups on the surface of the materials. The results for CAU-10-H, C-MDC, Al-fu, and A-MDC are shown in Figure 6. As shown in Figure 6a, various peaks were observed for CAU-10-H. The peak at 3621 cm^−1^ corresponds to μ-OH vibrations of the aluminum oxide (AlO_6_) clusters and water, whereas the peaks at 1558 cm^−1^ and 1403 cm^−1^ correspond to the asymmetric and symmetric vibrations of the coordination carboxylate groups, respectively [10,46]. In contrast, no peaks were observed for C-MDC, which indicates the formation of a hydrophobic carbon material without any functional groups on the surface.

Various peaks were also observed for Al-fu (Figure 6b). The peak at 3706 cm^−1^ corresponds to the μ-OH vibrations of the aluminum oxide (AlO_6_) clusters and water, whereas the peaks at 1596 cm^−1^ and 1423 cm^−1^ correspond to the asymmetric and symmetric stretching of carboxylic groups, respectively. The peak at 987 cm^−1^ corresponds to O-H bending [47,48,49]. Similar to that in C-MDC, the absence of peaks in the FTIR results of A-MDC after chemical treatment suggests the presence of a hydrophobic carbon material.

The structural defects and crystallinity of the carbon materials were determined from Raman spectroscopy results. The D band (1320 cm^−1^) corresponds to structural defects or amorphous carbon, whereas the G band (1590 cm^−1^) corresponds to graphitic ordered carbon. The ratio I_D_/I_G_, which represents the intensity of the D band normalized to that of the G band, indicates the presence of structural defects in carbon. A higher I_D_/I_G_ ratio suggests a higher level of structural imperfections [50,51,52]. The I_D_/I_G_ ratio of A-MDC is higher than that of C-MDC. This implies that the structural changes in C-MDC are minimal, even after thermal and chemical treatments, whereas the structural changes in A-MDC are expected to be considerable. These structural defects may influence the pore structure of the carbon materials, suggesting that A-MDC may have larger pores than C-MDC.

**Figure 7:**
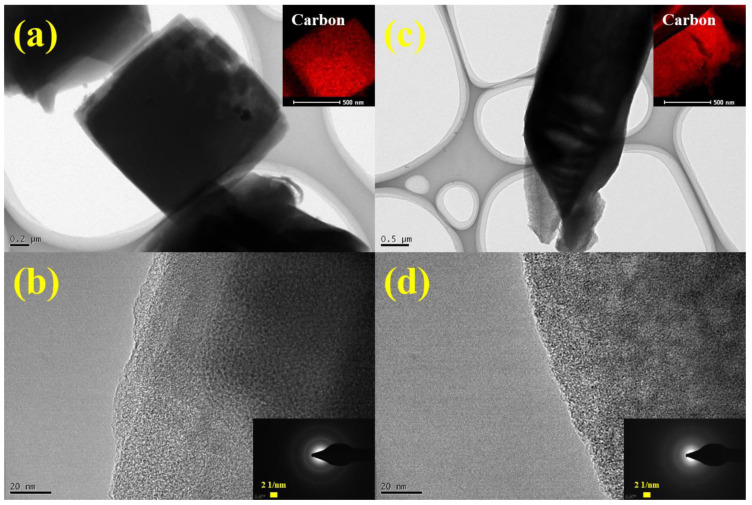
Results of TEM analysis. TEM images of C-MDC (**a**,**b**) and A-MDC (**c**,**d**). The insets show the EDS mapping and SAED pattern.

The TEM images and results of EDS mapping revealed that both synthesized C-MDC and A-MDC predominantly consisted of carbon, which is consistent with the particle size observed in SEM analysis (Figure 7a,c). The SAED patterns, which are in good agreement with the XRD results, further confirm the presence of amorphous carbon in both the MDC materials (Figure 7b,d).

**Figure 8:**
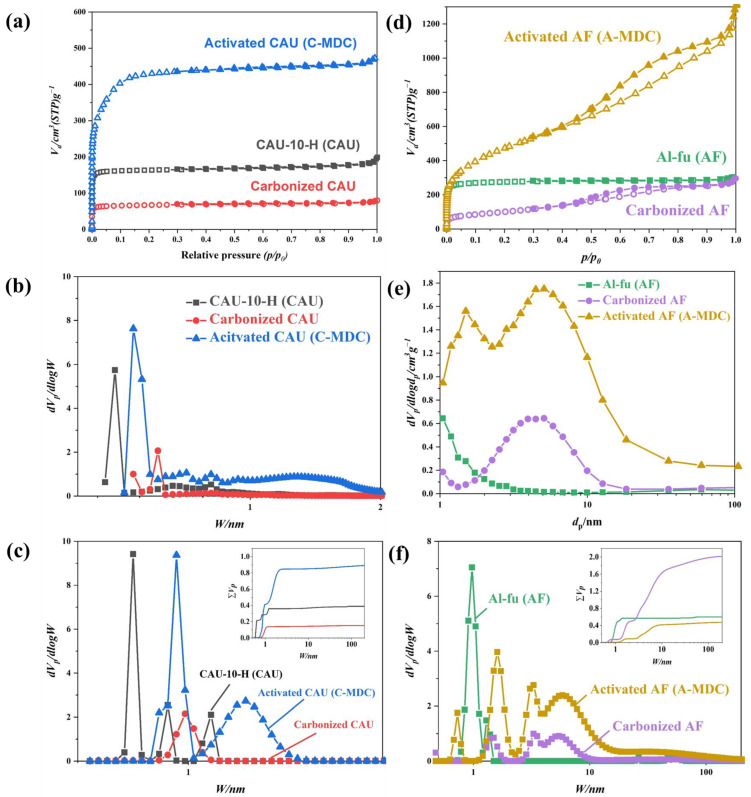
N_2_ adsorption and desorption isotherm at 77 K. Isotherm adsorption and desorption curve (**a**); HK Plot (**b**); NLDFT calculation (**c**) of CAU-10-H and its derivatives, isotherm adsorption and desorption curve (**d**); BJH Plot (**e**); NLDFT calculation (**f**) of Al-fu and its derivatives.

Figure 8a–c show the results of the isothermal nitrogen adsorption and desorption analyses for CAU-10-H, Al-fu, and its derivatives. The specific surface areas, pore sizes, and pore volumes of CAU-10-H and its derivatives are summarized in Table 5. As shown in Figure 8a, both CAU-10-H and C-MDC exhibit adsorption characteristics at relative pressures below 0.05, indicating microporous characteristics. The nitrogen adsorption capacity decreased during carbonization because pure aluminum metal and other impurities produced by the decomposition of aluminum clusters and organic ligand linkers during carbonization occupy pores of the structure, thereby blocking the pores. In contrast, the nitrogen adsorption capacity increased significantly after activation because the metallic and impurity components that blocked the pores were removed by the alkaline NaOH solution. Compared to the precursor, C-MDC exhibited a considerably higher nitrogen adsorption capacity, which is primarily attributed to the additional surface area resulting from the removal of aluminum clusters. This hypothesis can be further supported by the Horvath–Kawazoe (HK) plot in Figure 8b. The pore size of CAU-10-H, as confirmed by the HK plot, was 1.708 nm, whereas that of C-MDC was 2.043 nm. Figure 8c shows the overall pore size distribution obtained from NLDFT calculations. The results further confirm the increase in pore size and volume after activation. Consequently, the specific surface area of C-MDC (1653.2 m^2^g^−1^) was approximately 2.5 times higher than that of the precursor (677.77 m^2^g^−1^).

The specific surface areas, pore sizes, and pore volumes of Al-fu, and its derivatives are summarized in Table 6. Similar to that observed for C-MDC, aluminum oxides produced during carbonization blocked the pores, leading to a decrease in overall pore characteristics (Figure 8d). However, in contrast to C-MDC, A-MDC exhibited a hysteresis loop and nitrogen adsorption characteristics at relative pressures of 0.5 or higher. The hysteresis loop is more evident once the impurities were removed via activation. This demonstrates mesoporous characteristics that were not observed in Al-fu, as indicated by the Barrett–Joyner–Halenda (BJH) plot shown in Figure 8e. The BJH plot shows that, compared to the precursor, the number of mesopores (4, 8 nm) increased considerably after carbonization and activation. Figure 8f shows the pore sizes and volumes obtained from NLDFT calculations. In A-MDC, the number of mesopores and micropores has significantly increased. Consequently, the BET (Brunauer, Emmett, and Teller) surface area (1736.6 m^2^g^−1^) of A-MDC produced through carbonization and activation was approximately 1.5 times higher than that of the precursor (1103.1 m^2^g^−1^).

The pore structures of the synthesized MDCs were significantly different despite the identical aluminum metal clusters of the CAU-10-H and Al-fu precursors. This difference can be attributed to the organic ligands of MOFs. Isophthalic acid, employed in the synthesis of CAU-10-H, is an organic ligand with a benzene ring. In contrast, fumaric acid, utilized in the synthesis of Al-fu, is an organic ligand with carbon double bonds but without a benzene ring. Hence, CAU-10-H retains its structural integrity even after heat treatment because the benzene rings are resistant to thermal degradation. In contrast, Al-fu, which comprises ligands with carbon double bonds, is susceptible to structural changes after heat treatment because carbon double bonds are susceptible to thermal cleavage. This difference is closely related to the cracking observed on the surface of A-MDC but not on the surface of C-MDC in the FE-SEM images.

**Figure 9:**
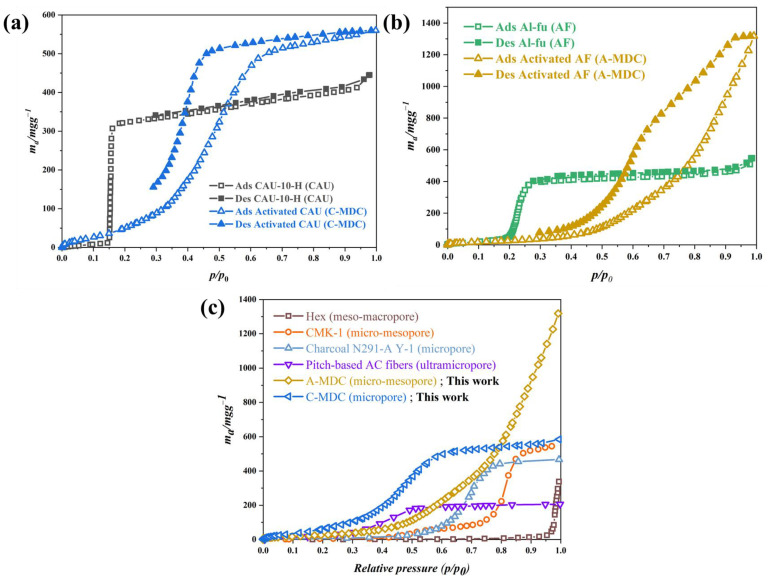
Water vapor adsorption and desorption isotherm curve at 298 K. CAU-10-H, C-MDC (**a**), Al-fu, A-MDC (**b**), compared with various carbon materials which have different sizes of pores (**c**) [27].

CAU-10-H and Al-fu MOF have been extensively studied as water adsorbents owing to their excellent stability and adsorption capacity for water. The water adsorption mechanism of carbonaceous materials is well known, and the water adsorption characteristics of MOFs and carbon materials are closely related to their pore structures and functional groups on the surface. CAU-10-H exhibited steeply increasing adsorption properties at relative pressures between 0.1 and 0.2 and no hysteresis loops in the adsorption and desorption curves. This suggests that the water adsorption mechanism of CAU-10-H is primarily influenced by its pore characteristics. However, the water adsorption properties of C-MDC were completely different. C-MDC exhibited continuous water adsorption at relative pressures below 0.3, with a remarkable increase in water adsorption around relative pressures of 0.4–0.6 (Figure 9a). The increase in water adsorption at relative pressures greater than 0.8 was not significant. This trend closely resembles the trends observed for type 5 carbon. Water adsorption by type 5 carbon occurs via (i) the adsorption of water molecules on functional groups on the carbon surface and the growth of clusters at low relative pressures (*P*/*P*_0_ > 0.3). This is due to the fact that as more water is adsorbed, individual water molecules begin to interact with each other by hydrogen boing, leading to the formation and growth of clusters. And (ii) the adsorption of water in micropore pores around relative pressures *P*/*P*_0_ < 0.8 [26]. In particular, there are differences in the adsorption range based on the size of micropores, and the synthesized C-MDC has pore sizes ranging from 1.1 nm to 2 nm. Therefore, it exhibits a rapid adsorption ability at relative pressures of 0.6 and above. Carbon materials with micropores smaller than 1.1 nm start adsorbing water at relative pressures below 0.6, but the quantity is not significant (Figure 9c). On the other hand, C-MDC with micropores larger than 1.1 nm demonstrates a higher adsorption capacity [27]. Hence, a limited number of functional groups might be present on the surface of C-MDC. These results can be predicted because most functional groups on the surface were removed by wet etching The hypothesis on the microporous characteristics was substantiated by analyzing the pore properties of C-MDC that were obtained from the nitrogen adsorption isotherm analyses.

Al-fu shows a steeply increasing water adsorption behavior in the relative pressure range of 0.2–0.3, similar to the behavior observed in CAU-10-H MOF, indicating that the water adsorption mechanism is governed primarily by pore characteristics. In contrast, the water adsorption of A-MDC is relatively low at relative pressures lower than 0.3, begins to increase at a relative pressure of 0.6, and increases significantly at relative pressures higher than 0.8 (Figure 9b). This behavior resembles the behavior observed for type 3 carbon. Type 3 carbon denotes hydrophobic carbon which exhibits (i) minimal water adsorption in the relative pressure range of *P*/*P*_0_ < 0.3 owing to the scarcity of functional groups on the surface; (ii) water adsorption in the micropores of the material in the relative pressure range of 0.6–0.8; and (iii) water adsorption in the mesopores at relative pressures *P*/*P*_0_ > 0.8. Moreover, the adsorption and desorption processes exhibit a hysteresis loop, which is a typical feature of mesoporous carbon materials and is attributed to capillary phenomena. Hence, it can be inferred that A-MDC possesses both micropores and mesopores, rendering it a hydrophobic carbon material, which agrees well with its previously described attributes.

Although the total amount of water adsorption is much higher in A-MDC than in C-MDC, the relative pressure range of adsorption is crucial for commercially used adsorbents. This is because the energy required for adsorption at different relative pressures varies considerably, which is important from an economic perspective. Therefore, C-MDC may be a better adsorbent candidate than A-MDC. Furthermore, C-MDC exhibits high water adsorption characteristics in the relative pressure range of 0.45–0.65, which is the desired range for indoor dehumidification adsorbents. Therefore, in this study, C-MDC was selected as the candidate water adsorbent. Additional water adsorption modeling simulations were conducted, and the adsorption enthalpy was calculated.

Comparing experimental and analytical data with existing theories increases the reliability of the data and is crucial for broader applications. Moreover, relying solely on a single standardized model to compare and predict the properties of a newly synthesized material may lead to significant errors. Therefore, two models related to the characteristics of pores and functional groups, which are important features of the synthesized samples, have been applied in this study.

**Figure 10:**
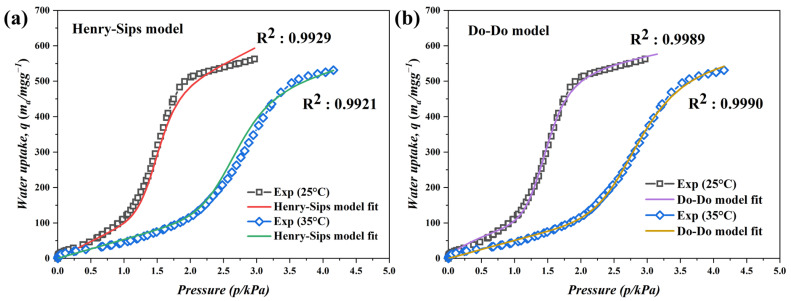
Equilibrium adsorption isotherm curves of water vapor on C-MDC. Symbols and lines represent experimental data and predicted isotherms data by Henry–Sips model (**a**) and Do–Do model (**b**), respectively.

The first model, known as the Henry–Sips model, is a hybrid adsorption model that combines the theories of two water adsorption mechanisms. The model equation includes parameters representing Henry’s law, which describes physical site adsorption from a thermodynamic perspective, and the Sips parameter, which represents adsorption by filling only the micropores. The parameters of the Henry–Sips model are presented in Table 7, and the analysis and modeling results at 298 K and 308 K are depicted in Figure 10a. Among the parameters, the value of K_s_ is larger than the values of β and K_H_. This is because the values of β and K_H_ are determined by Henry’s law, which represents adsorption by functional group sites. However, the value of K_s_ is determined by the Sips law, which represents adsorption due to the filling of micropores. The Henry–Sips model exhibits a good fit, with R^2^ values of 0.9929 and 0.9921 at 298 K and 308 K, respectively.

The second model, known as the Do–Do model, demonstrates excellent compatibility of water adsorption for type 5 carbons, which are hydrophobic microporous carbon materials. Similar to the Henry–Sips model, the Do–Do model suggests that the initial site adsorption occurs on the functional groups, followed by a sharp increase in adsorption at high pressure through capillary condensation. The parameters of the Do–Do model are listed in Table 8, and the analysis and modeling results at 298 K and 308 K are compared in Figure 10b. In the Do–Do model, ***f*** and K_f_ represent the adsorption at the sites, while K_μ_, α, and β represent the pore adsorption. Hence, the values of ***f*** and K_f_ are small enough to be ignored, whereas the values of K_μ_, α, and β related to pore adsorption are relatively large. This observation adequately explains the water adsorption characteristics of hydrophobic C-MDC. The Do–Do model exhibits excellent compatibility, with R^2^ values of 0.9989 and 0.9990 at 298 K and 308 K, respectively. Therefore, it can be concluded that the C-MDC synthesized in this study possesses properties that closely resemble those of hydrophobic microporous materials.

**Figure 11:**
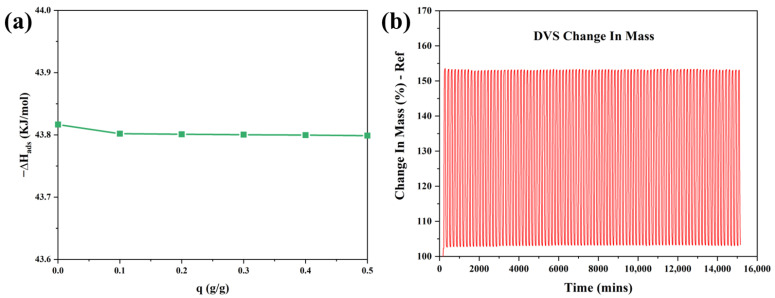
Isosteric adsorption enthalpy (**a**) and the adsorption and desorption cycle test to assess the stability of adsorption on C-MDC (**b**).

The heat of adsorption is a crucial factor in process design. The isosteric adsorption enthalpy of C-MDC was calculated based on the water adsorption data at 298 K and 308 K using the Clausius–Clapeyron equation, and the results are shown in Figure 11a. The initial adsorption enthalpy was −43.81 kJ/mol and did not change considerably with an increase in adsorption. Typically, values in the range of −80 to −400 kJ/mol indicate chemical adsorption, whereas values greater than −80 kJ/mol suggest physical adsorption [53]. Hence, it can be inferred that the water adsorption by C-MDC is predominantly physical, which supports the previously described mechanism wherein water adsorption is primarily attributed to physical adsorption.

Water stability is an important factor for adsorbents. The water stability of C-MDC was analyzed via dynamic vapor sorption analysis by measuring the water sorption and desorption cycles in the *P*/*P*_0_ range from 10–90 (Figure 11b). The changes in weight during the first and 100th cycles were 51.14% and 50.04%, respectively (Table 9). It was confirmed that 97.84% of the initial water sorption was maintained over 100 cycles, and the difference between the kinetics during the initial and subsequent cycles was not significant. This confirms the stability and consistency of the water sorption and desorption capacities of C-MDC. These results are considered valuable because water stability is a crucial factor for potential commercial applications of C-MDC.

## 4. Conclusions

In this study, a comprehensive investigation was conducted to examine the pore characteristics of MDCs synthesized via carbonization and activation. The influence of various ligands used in the construction of aluminum-based organic/inorganic nanoporous materials (MOFs) was investigated. CAU-10-H, synthesized with isophthalic acid ligands containing benzene rings, maintains its structure without significant changes even after thermal treatment because of the preservation of its bonds. The removal of the aluminum clusters creates additional space, allowing for the expansion of the size and volume of the micropores. In contrast, Al-fu prepared using fumaric acid ligands containing double bonds exhibits structural changes during thermal treatment. The relatively weak carbon bonds break and reform, leading to the formation of mesopores which are absent in the MOF precursor. This results in a significant increase in pore volume and specific surface area. Based on these results, the microporous C-MDC was evaluated as a water adsorbent and isothermal water adsorption modeling was explored for extended usage. With its high water adsorption capacity in the relative pressure range of 45–65%, C-MDC demonstrates the potential for utilization as an indoor humidity control desiccant. In addition, the simulation results of the Do–Do model, specifically optimized for type 5 adsorption, showed excellent compatibility. The large-scale synthesis of MDC based on MOFs proposed in this study has the potential to guide the applications of MDC as water adsorbents.

## Figures and Tables

**Table 1:** Elemental composition of CAU-10-H and its derivatives analyzed via XPS.

Samples (at. %/XPS)	C1s	O1s	Al2p	Total
CAU-10-H (CAU)	72.75	23.49	3.77	100
Carbonized CAU	69.23	20.2	10.55	100
Activated CAU (C-MDC)	90.35	9.65	0	100

**Table 2:** Elemental composition of Al-fu and its derivatives analyzed via XPS.

Samples (at. %/XPS)	C1s	O1s	Al2p	Total
Al-fu (AF)	58.98	33.59	7.44	100
Carbonized AF	46.88	33.45	19.68	100
Activated AF (A-MDC)	89.32	10.68	0	100

**Table 3:** Elemental composition of CAU-10-H and its derivatives analyzed via EA and EDS.

**Samples (wt.%/EA)**	**C**	**O**	**N**	**H**	**Total**
CAU-10-H (CAU)	45.44 (±0.01)	27.31 (±0.18)	0	2.66 (±0.03)	75.41
Carbonized CAU	41.66 (±0.09)	6.17 (±0.23)	0	0.83 (±0.06)	48.67
Activated CAU (C-MDC)	91.57 (±0.08)	3.88 (±0.08)	0	0.65 (±0)	96.10
**Samples (wt.%/EDS)**	**C**	**O**	**N**	**Al**	**Total**
CAU-10-H (CAU)	66.61	30.12	0	3.27	100
Carbonized CAU	56.89	32.79	0	10.32	100
Activated CAU (C-MDC)	86.48	13.52	0	0	100

**Table 4:** Elemental composition of Al-fu and its derivatives analyzed via EA and EDS.

**Samples (wt.%/EA)**	**C**	**O**	**N**	**H**	**Total**
Al-fu (AF)	29.90 (±0.05)	44.07 (±0.74)	0	1.96 (±0.09)	75.93
Carbonized AF	25.91 (±0.02)	7.74 (±0.30)	0	0.92 (±0.07)	34.34
Activated AF (A-MDC)	90.30 (±0.13)	5.17 (±0.29)	0.17 (±0.00)	1.07 (±0.00)	96.72
**Samples (wt.%/EDS)**	**C**	**O**	**N**	**Al**	**Total**
Al-fu (AF)	29.84	52.32	0	17.84	100
Carbonized AF	37.80	40.10	0	22.10	100
Activated AF (A-MDC)	88.27	11.73	0	0	100

**Table 5:** BET surface area, pore size, and pore volume of CAU-10-H and its derivatives.

	S_BET_ [m^2^g^−1^]	V_total_ [cm^3^g^−1^]	D_ave_ [nm]
CAU-10-H (CAU)	677.77	0.2894	1.7078
Carbonized CAU	241.51	0.1234	2.0430
Activated CAU (C-MDC)	1653.2	0.7291	1.7640

**Table 6:** BET surface area, pore size, and pore volume of Al-fu and its derivatives.

	S_BET_ [m^2^g^−1^]	V_total_ [cm^3^g^−1^]	D_ave_ [nm]
Al-fu (AF)	1103.1	0.4664	1.6911
Carbonized AF	365.37	0.4328	4.7385
Activated AF (A-MDC)	1736.6	1.8394	4.2366

**Table 7:** The Henry–Sips model fitting parameters.

Henry–Sips	β	K_H_	q_m_	K_s_	A	B
	0.6594	0.4568	0.9141	2.0937	0.0099	0.2897

**Table 8:** The Do–Do model fitting parameters.

Do–Do	*f*	K_f_	K_μ_	α	β
298 K	0.0383	0.0063	0.1058	0.6914	10.769
308 K	0.0388	2.9530 × 10^−5^	0.0556	1.0466	9.6265

**Table 9:** Changes in water adsorption and desorption after cycle test for C-MDC.

Change in Mass (%)		Target	C-MDC
Desorption	Sorption	*P*/*P*_0_ (%)	
2.66	2.38	10.0	Cycle 1
53.52	53.52	90.0	
3.37	3.07	10.0	Cycle 100
53.11	53.11	90.0	

## Data Availability

Not applicable.

## Supplementary Materials

The following supporting information can be downloaded at: https://www.mdpi.com/article/10.3390/nano13162351/s1, S.1. Large-scale synthesis of aluminum fumarate MOF (Al-fu). S.2. Large-scale synthesis of A-MDCs; Figure S1: 100 L reflux reactor used for large-scale synthesis of Al-fu; Figure S2: HRTF-180/60 (HANTECH, Korea) rotary furnace used for large-scale carbonization of Al-fu; Figure S3: Characteristics of large-scale synthesized Al-fu MOF. XRD pattern (a), FE-SEM image (b), nitrogen adsorption isotherm curve at 77 K (c), and NLDFT calculation (d); Figure S4: Characteristics of carbonized AF and activated AF (A-MDC) (50 g of Al-fu MOF placed in the rotary furnace). XRD pattern (a) and FE-SEM image (b) of carbonized AF, XRD pattern (c) and FE-SEM image (d) of activated AF (A-MDC); Figure S5: Nitrogen adsorption isotherm of activated AF (A-MDC) (50 g of Al-fu MOF placed in the rotary furnace). Isotherm curve at 77 K (a), NLDFT calculation (b), and BJH plot (c); Figure S6: Characteristics of carbonized AF and activated AF (A-MDC) (300 g of Al-fu MOF placed in the rotary furnace). XRD pattern (a) and FE-SEM image (b) of carbonized AF, XRD pattern (c) and FE-SEM image (d) of activated AF (A-MDC); Figure S7: Nitrogen adsorption isotherm of activated AF (A-MDC) (300 g of Al-fu MOF placed in the rotary furnace). Isotherm curve at 77 K (a), NLDFT calculation (b), and BJH plot (c); Figure S8: Nitrogen adsorption isotherm of activated AF (A-MDC) (600 g of Al-fu MOF placed in the rotary furnace). Isotherm curve at 77 K (a), NLDFT calculation (b), and BJH plot (c); Table S1: BET surface area, total pore volume, and average pore size of large-scale synthesized Al-fu, and its derivatives; Figure S9: The structure of CAU-10-H; Figure S10: The structure of Al-fu MOF.
