# Supplementary material for: Synthesis of Aluminum-Based Metal–Organic Framework (MOF)-Derived Carbon Nanomaterials and Their Water Adsorption Isotherm
Source: Nanomaterials (Basel). 2023 Aug 16;13(16):2351. doi: 10.3390/nano13162351 (PMC10458837; doi:10.3390/nano13162351)
Supplement: Supplementary file 1 [file nanomaterials-13-02351-s001.zip › nanomaterials-2540199-Supplementary.pdf]

MDPI

nanomaterials

Supplementary Materials

Synthesis of Aluminum-Based Metal–Organic Framework (MOF)-Derived Carbon Nanomaterials and Their Water Adsorption Isotherm

Dasom Jeong [1,2], Seong Cheon Kim [1,3], Taeseop An [1], Dongho Lee [4], Haejin Hwang [2], Siyoung Q. Choi [3,5] and Jeasung Park [1,*]

[1] Green and Sustainable Materials R&D Department, Korea Institute of Industrial Technology (KITECH), Cheonan 31056, Republic of Korea; jdasom0130@kitech.re.kr (D.J.); grey@kitech.re.kr (S.C.K.); ats2660@kitech.re.kr (T.A.)
[2] Department of Materials Science and Engineering, INHA University, Incheon 22212, Republic of Korea; hjhwang@inha.ac.kr
[3] Department of Chemical and Biomolecular Engineering, Korea Advanced Institute of Science and Technology (KAIST), Daejeon 34141, Republic of Korea; sqchoi@kaist.ac.kr
[4] Process R&D Center, Hanwha Solutions R&D Institute, Daejeon 34128, Republic of Korea; dhlee@hanwha.com
[5] KAIST Institute for the Nanocentury, Korea Advanced Institute of Science and Technology (KAIST), Daejeon 34141, Republic of Korea
* Correspondence: jpark@kitech.re.kr

S.1. Large-scale synthesis of aluminum fumarate MOF (Al-fu)

15.728 kg of aluminum sulfate and 4.496 kg of urea were added into a 100 L reactor containing 80 L of deionized water and stirred until fully dissolved. Subsequently, 5.792 kg of fumaric acid was added, and the mixture was stirred for 60 min at room temperature. The mixture was then refluxed at 80°C for 24 h followed by refluxing at 100°C for another 24 h. The resulting solution was washed three times with deionized water, filtered, and dried in vacuum oven at 80°C overnight to obtain Al-fu. (Quantity:6.79 kg, yield:85%)

S.2. Large-scale synthesis of A-MDCs

Large-scale synthesized Al-fu was used as a precursor to produce MDCs. An HRTF-180/60 (HANTECH, Korea) rotary furnace with a tube size of 180 × 60 × 520 mm was used for carbonization, and nitrogen gas (99.999%) was supplied at a flow rate of 200 cc/min. Sodium hydroxide (98.0%, SAMCHUN, Korea) was used as the wet etching solution for the carbonized Al-fu.

To prepare A-MDC, 50, 300, and 600 g of Al-fu were placed in a rotary furnace tube. The sample was then carbonized at 800°C for 3 h under a nitrogen atmosphere. After cooling to room temperature, the carbonized sample was poured in 10M sodium hydroxide solution and activated by stirring for 1 h at 90°C. The activated sample was washed thoroughly with deionized water, filtered, and dried overnight in a vacuum oven at 80°C to obtain the samples.

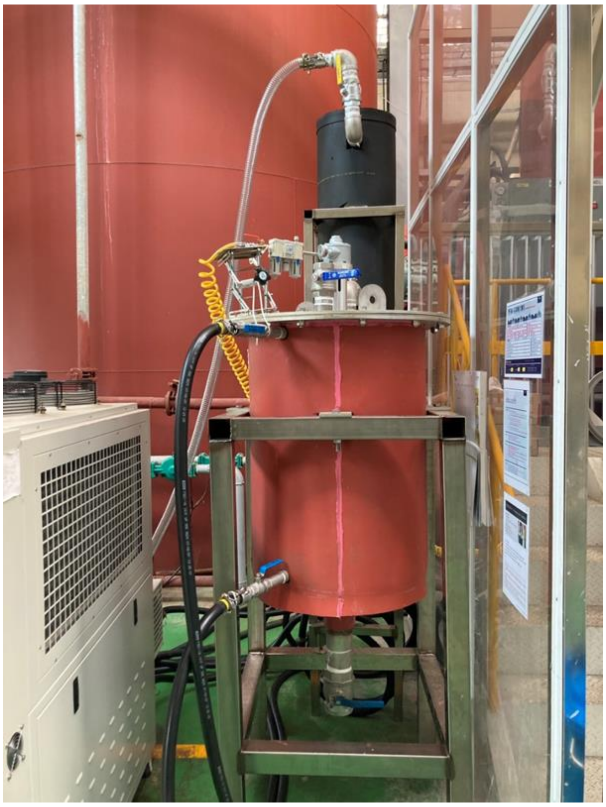

Figure S1. 100L reflux reactor used for large-scale synthesis of Al-fu.

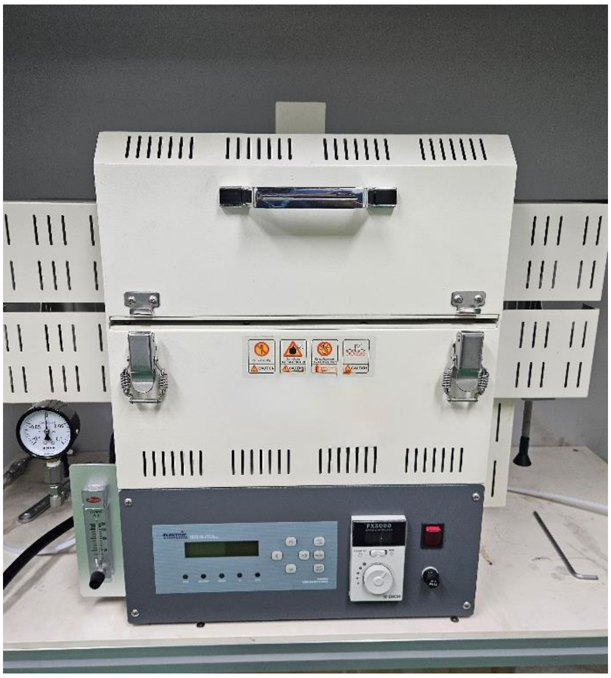

Figure S2. HRTF-180/60 (HANTECH) rotary furnace used for large-scale carbonization of Al-fu.

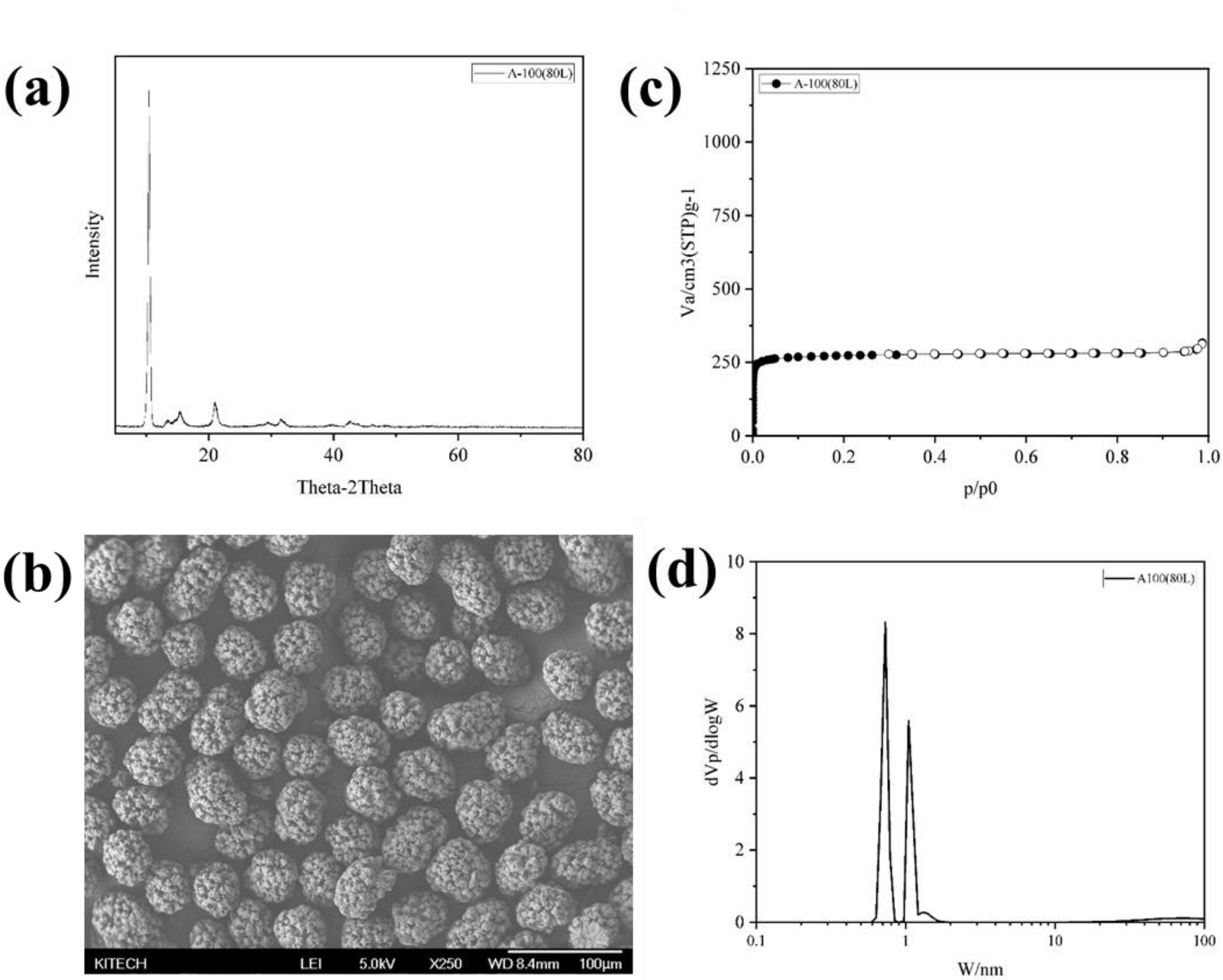

Figure S3. Characteristics of large-scale syntheized Al-fu MOF. XRD pattern (a), FE-SEM image (b), Nitrogen adsorption isotherm curve at 77K (c), and NLDFT calculation (d).

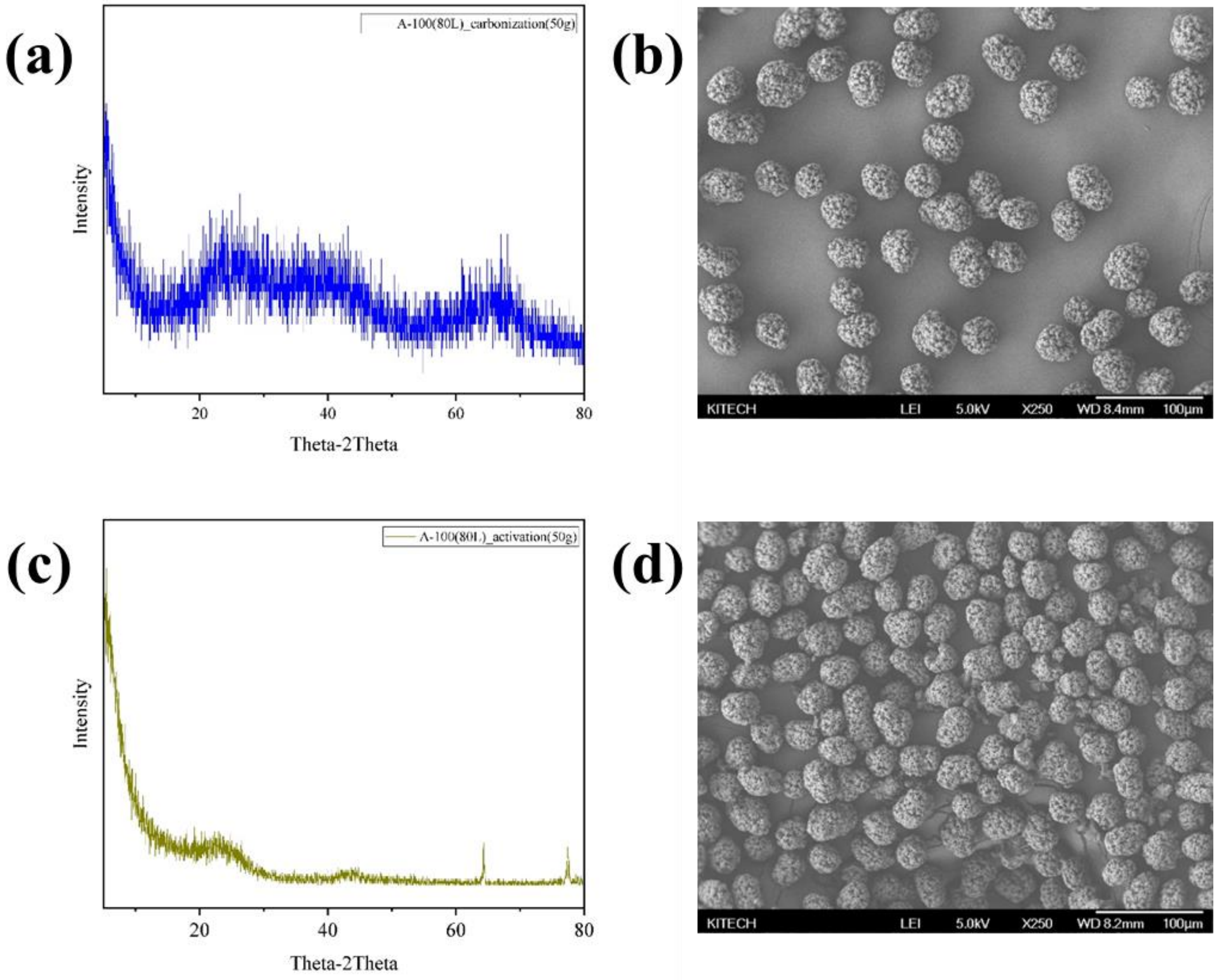

Figure S4. Characteristics of carbonized AF and activated AF (A-MDC) (50g of Al-fu MOF placed in the rotary furnace). XRD pattern (a) and FE-SEM image (b) of carbonized AF, XRD pattern (c) and FE-SEM image (d) of activated AF (A-MDC).

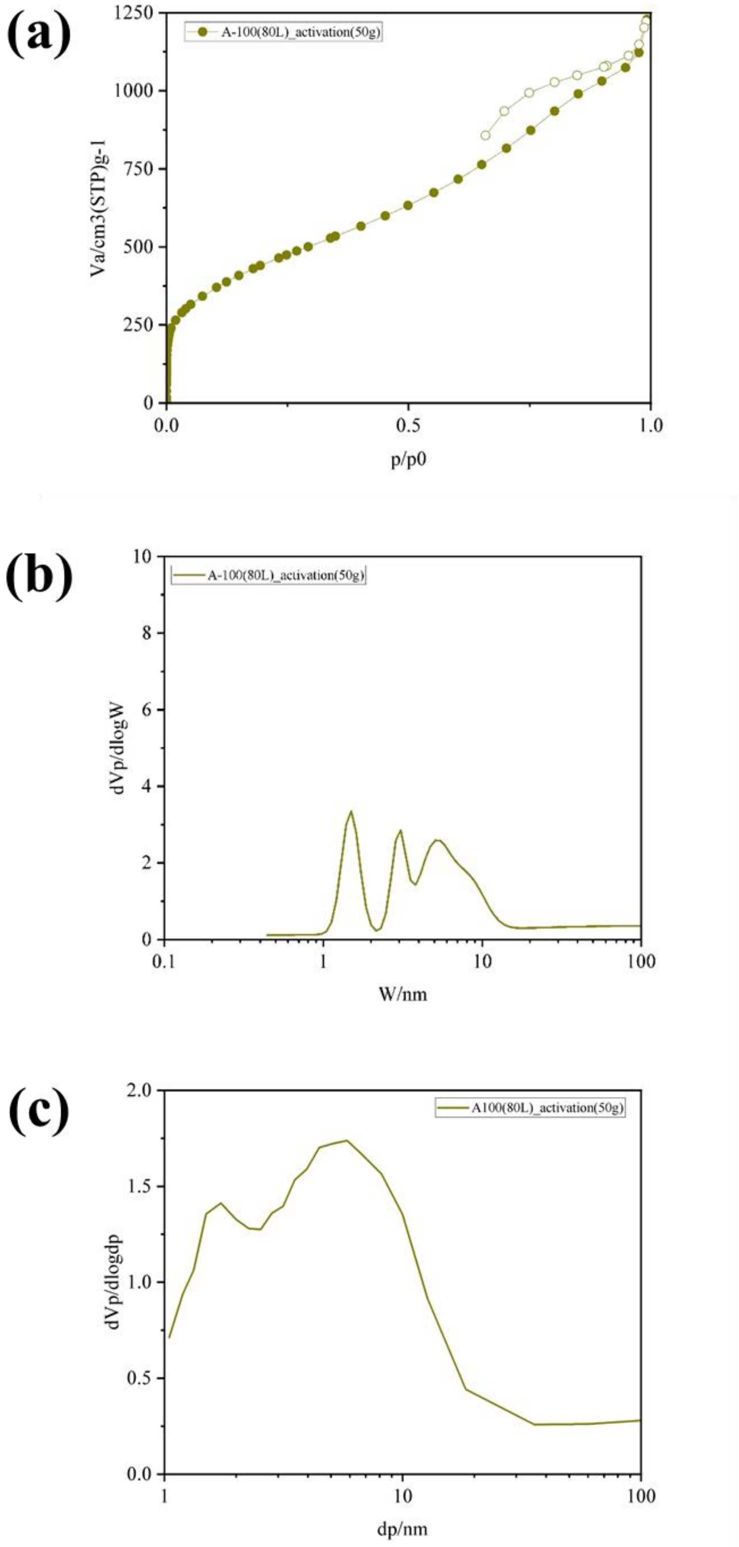

Figure S5. Nitrogen adsorption isotherm of activated AF (A-MDC) (50g of Al-fu MOF placed in the rotary furnace). Isotherm curve at 77 K (a), NLDFT calculation (b), and BJH plot (c).

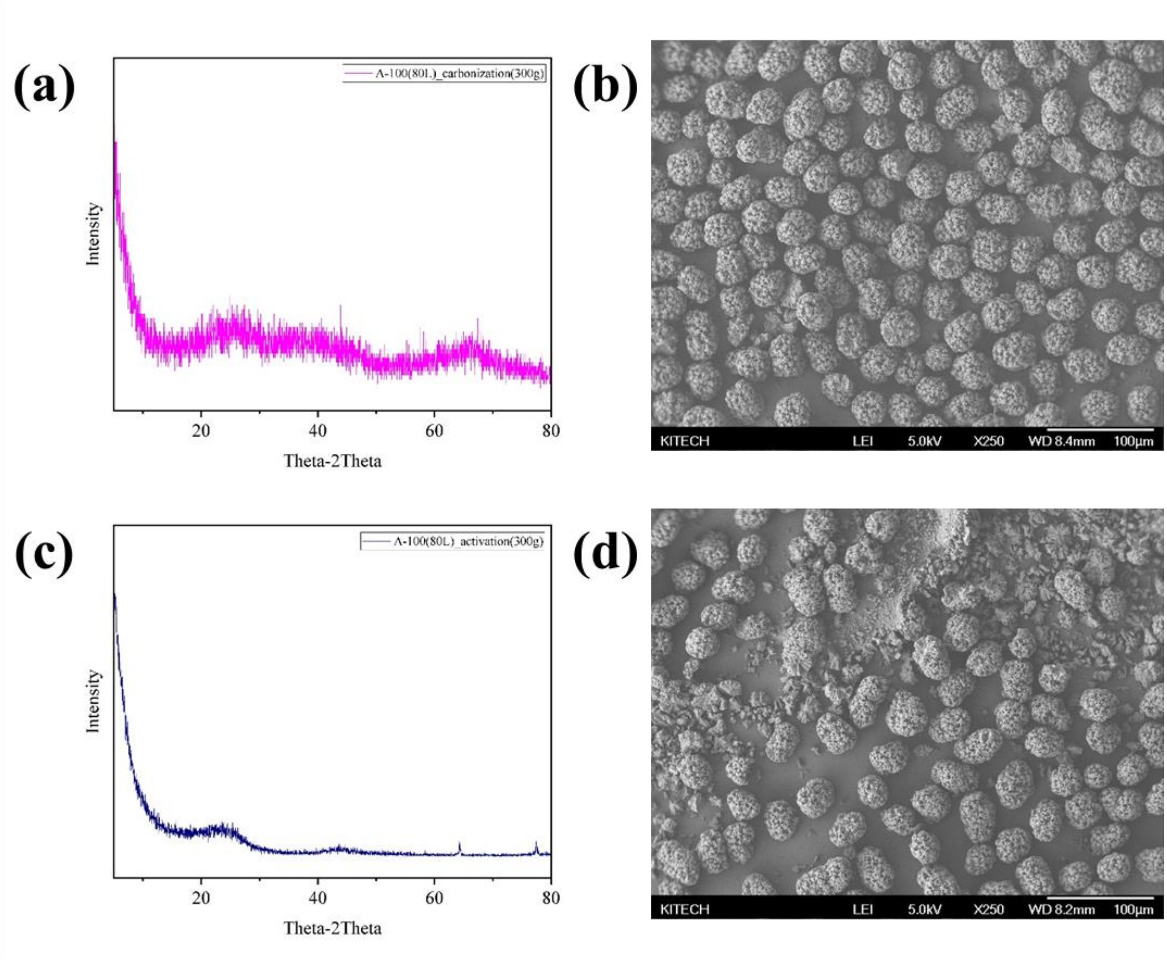

Figure S6. Characteristics of carbonized AF and activated AF (A-MDC) (300g of Al-fu MOF placed in the rotary furnace). XRD pattern (a) and FE-SEM image (b) of carbonized AF, XRD pattern (c) and FE-SEM image (d) of activated AF (A-MDC).

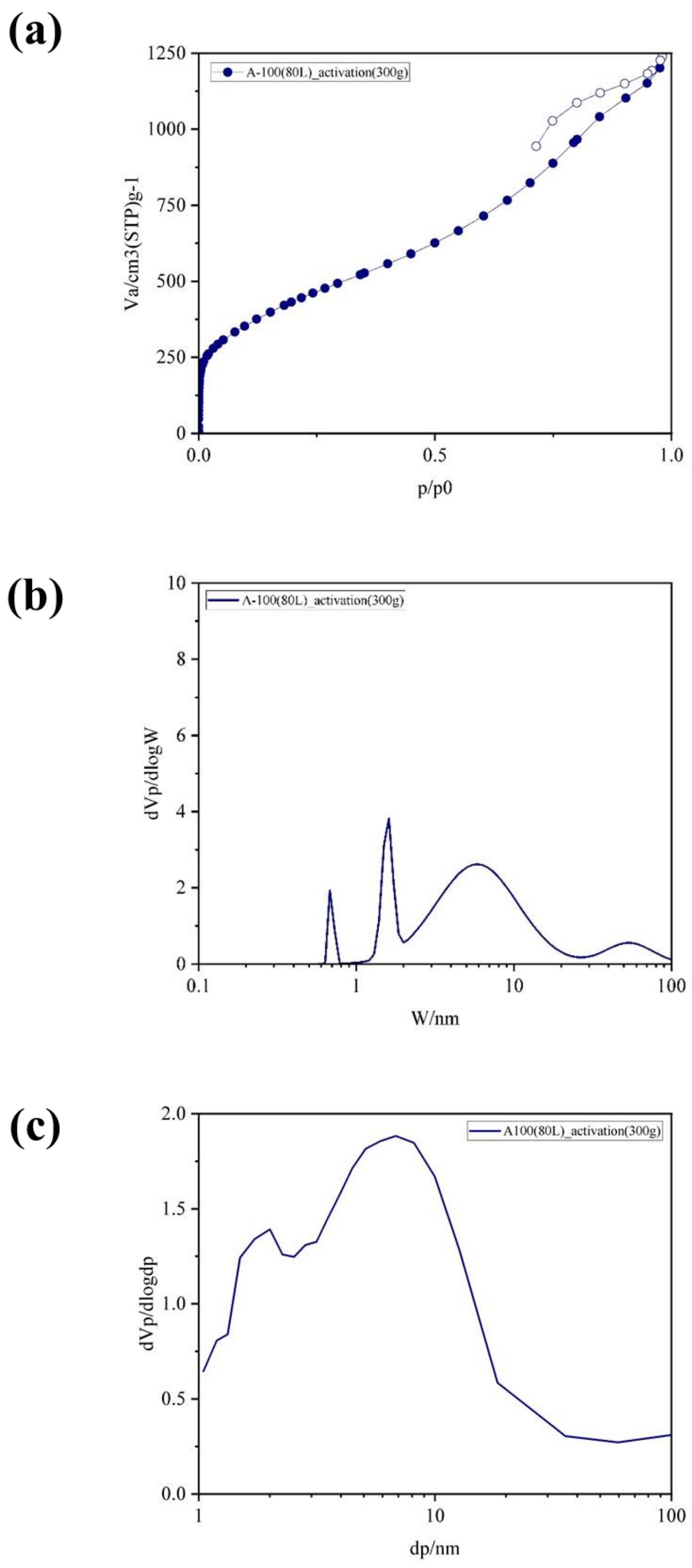

Figure S7. Nitrogen adsorption isotherm of activated AF (A-MDC) (300g of Al-fu MOF placed in the rotary furnace). Isotherm curve at 77 K (a), NLDFT calculation (b), and BJH plot (c).

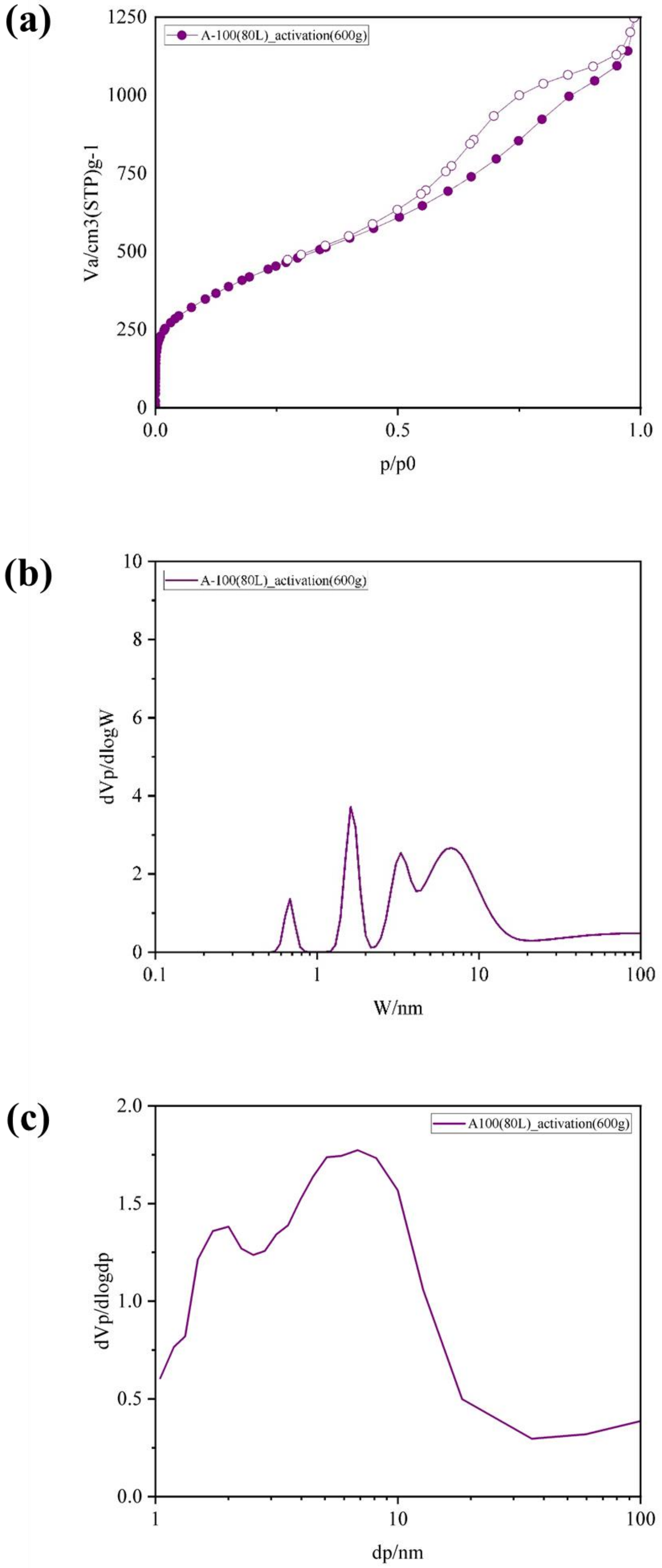

Figure S8. Nitrogen adsorption isotherm of activated AF (A-MDC) (600g of Al-fu MOF placed in the rotary furnace). Isotherm curve at 77 K (a), NLDFT calculation (b), and BJH plot (c).

Table S1. BET surface area, total pore volume, and average pore size of large-scale synthesized Al-fu, and its derivatives.

	S_{BET} [m^2g^{-1}]	V_{total} [cm^3g^{-1}]	D_{ave} [nm]
Large scale synthesized Al-fu (AF)	1079	0.4893	1.814
Activated AF (A-MDC) (50g)	1567.4	1.8745	4.7837
Activated AF (A-MDC) (300g)	1551	1.9590	5.0523
Activated AF (A-MDC) (600g)	1576.7	1.9394	4.9201

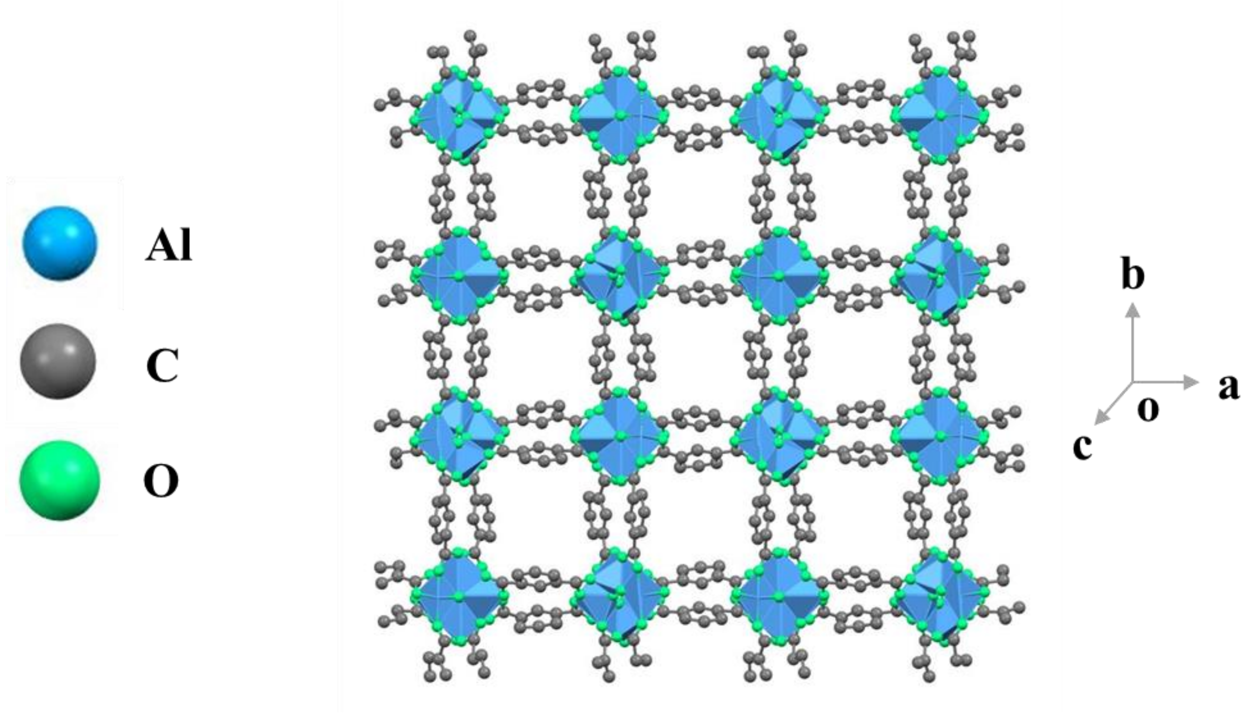

Figure S9. The structure of CAU-10-H.

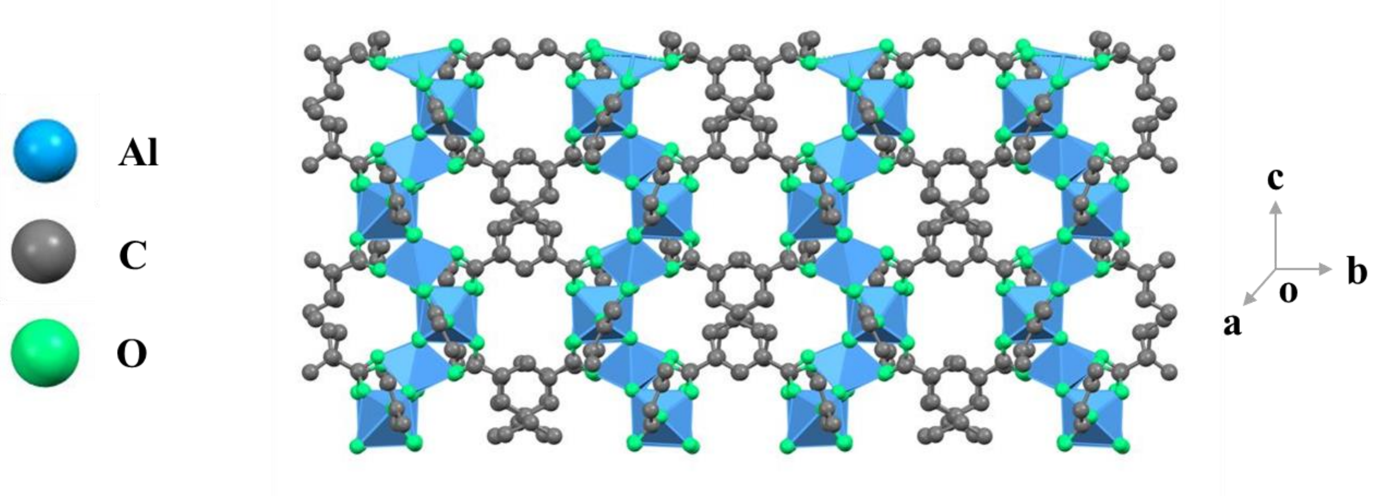

Figure S10. The structure of Al-fu MOF.
